# Treatment Seeking Pattern Among Parents of Children with Locomotor Disablity

**DOI:** 10.4103/0970-0218.55297

**Published:** 2009-07

**Authors:** Ananya Ray Laskar, VK Gupta, MM Singh, Dharmendra Kumar, GK Ingle

**Affiliations:** Department of Community Medicine, Maulana Azad Medical College, New Delhi, India; 1 Department of Community Medicine, PDU Institute for the Physically Handicapped, New Delhi, India

## Introduction

Locomotor disability accounts for the highest number (1046) in rural and (109) in urban for every 100,000 population out of disabilities, according to NSSO.(1) Inspite of these figures, there is poor dissemination of information regarding specialized care for the locomotor disabled children. This leads to a substantial amount of ambiguity among caregivers in relation to which healthcare facility is to be consulted first. Moreover, there is no single point of entry into the multiple systems of care.(2) Often, complex criteria determine the availability or referral of services and delay detection and commencement of treatment. Socio-economic and cultural barriers further worsen the prognosis of the case.(3) Addressing the unmet health needs may improve the health-seeking behavior and thereby disease outcomes.

## Materials and Methods

A cross-sectional study was conducted in the OPD of Deen Dayal Upadhyay Institute for Physically Handicapped (IPH), Vishnu Digambar Marg, New Delhi, from April 2005 to March 2006. All subjects in the age group 6-15 years attending the OPD during this period fulfilled the inclusion criteria and those willing to participate were enrolled in the study.

Inclusion criteria: (i) children in the age group of 6-15 years, with permanent locomotor disability (ii) those accompanied by atleast one of the two parents.

Exclusion criteria: (i) Children with multiple disabilities such as visual, hearing or mental disability along with locomotor disability. (ii) Not staying with any of the parents.

Parents were interviewed using a semi-structured pre-tested questionnaire in order to elicit treatment- seeking pattern. The questionnaire included information about the place, nature and time of first and subsequent consultations, reasons for choosing the place of consultations, time period between onset of symptoms and first consultation, time gap between first consultation and commencement of treatment as perceived by the parent, treatment or rehabilitative services received at each consultation. The present and previous treatment records were also tallied to get additional information.

The study used the Government of India. definition - Locomotor Disability is a person's inability to perform distinctive activities associated with moving both himself and objects from place to place and such inability resulting from affliction of either bones, joints, muscles or nerves depending on the extent.(4)

## Results

Of the total 329 cases in this age group attending the OPD of IPH during the study period, 100 were selected based on the completeness of records, fulfillment of inclusion criteria and willingness to participate, 58% of the subjects were of urban origin and belonged to Hindu nuclear families, 50% of them belonged to the upper-lower class and the rest belonged to lower-middle (33%) or lower class (17%). Overall, 76% of the subjects had already consulted three/four health care facilities at time of the study, maximum used were six.

Initially, consultations with Government Hospitals were only 22% as depicted in Table 1. Rehabilitative Institutions such as IPH, providing specialized care such as occupational therapy or physiotherapy, aids and appliances and psychological counseling services were rarely consulted in the initial few consultations. About 40% parents approached private hospitals or clinics. The most common reasons cited were- long hours in queue (57%), ill-treatment by staff especially those relying on aids and appliances (45%), complicated paper work (36%) and overall poor quality of care (28%) in Government set-up. Besides, institutions providing specialized care were only a few in their knowledge and inaccessible to them.

**Table 1:** Pattern of first consultations and subsequent consultations

Type of health care facility	Order of consultation				
	
	I^st^	II^nd^	III^rd^	IV^th^	V^th^
Alternative med*.	21	7	18	15	56
Govt. hosp.	22	61	53	12	4
Pvt. hosp.	40	19	10	22	0
Quack	15	1	0	4	3
Others**	2	11	3	6	1
IPH	0	1	15	41	36

* Include- Ayurvedic, Homeopathy, Unani and traditional Medicine practitioners,

** NGOs, Dispensaries, and PHCs

The percentage of parents of disabled children who approached the Government hospital after having sought treatment elsewhere increased to 61%. There is also a sharp fall in the proportion of people attending the private hospital or clinic. A substantial rise is evident in the number of parents consulting specialized centers, such as IPH.

Table 2 shows 55% of the subjects from rural background consulted the alternate system of medicine for the first time. While urban residents preferred attending the private health care facility (34%), the quacks were more popular (30%) amongst the urban slum dwellers as shown in Table 2. A significant association was found between the type of residence with the first healthcare facility consulted, with an Odds Ratio of 6.28 (95% CI = 2.34 to 17.22), χ^2^ value of 17.39 and *P* value of 0.0003.

**Table 2:** First health care facility consulted by various sections

Type of residence	First health care facility					Total
		
	Alt med*	Govt. hosp.	Pvt. hosp.	Quack	Others	
Rural n (%)	5 (55.6)	2 (22.2)	0	2 (22.2)	0	9 (100)
Urban n (%)	8 (13.8)	13 (22.4)	34 (58.6)	3 (5.2)	0	58 (100)
Urban slum n (%)	8 (22.2)	7 (22.2)	6 (18.2)	10 (30.3)	2 (6.1)	33 (100)

* Include- Ayurvedic, Homeopathy Unani and traditional Medicine practitioners,

** OTHERS here, Rural Dispensaries, PHCs and NGOs

From Table 3 it is clear that parents of subjects belonging to the lower socio-economic status had the most faith on alternate system of medicine (39.4%), but private facilities were far more popular among the lower-middle (70.6%) and even the upper lower (44%) strata. Unfortunately, 68% of these private practitioners were just general physicians. There was significant statistical association of the socio- economic status with the place of first consultation with an OR =0.24 (95% C.I 0.09-0.63) χ^2^ =of 10.68 and *P* value of 0.00108.

**Table 3:** First consultation by families from different socio-economic status

Socio-economic status	First health care facility					Total
		
	Alt med*	Govt hosp.	Pvt hosp.	Quack	Others	
Lower n (%)	13 (39.4)	7 (21.2)	6 (18.2)	5 (15.2)	2 (6.1)	33 (100)
Upper-Lower n (%)	8 (16.0)	10 (20.0)	22 (44.0)	10 (20.0)	0	50 (100)
Lower-Middle n (%)	0	5 (29.4)	12 (70.6)	0	0	17 (100)

Around 50% of parents of study subjects consulted the healthcare facility within few hours of the onset of the disease, followed by 34% who attended after one/two days, as given in Table 4. Only two per cent of them consulted after a month of onset. The median time lapse between onset of disease and first consultation was six hours with inter-quartile range of immediately to 24 hours.

**Table 4:** Time lapse between onset of disease with first consultation and commencement of treatment

Time lapse between onset of disease	First consultation (%)	Commencement of treatment (%)
Immediately/few hrs	50	4
1-2 days	34	6
>2 days to 1 week	8	17
>1 week - 1 month	4	22
>1 month - 6 months	2	31
>1 year	2	20

Only in four per cent, treatment was started immediately or within few hours, as perceived by the parents. In majority of the cases the treatment was started within one to six months (31%). The median time lapse between onset of disease and commencement of the treatment was one month with an inter-quartile range of one week to six months.

## Discussion

The pattern of first consultation highlights the fact that parents of disabled children have little faith in the government health care system and prefer consulting the private clinic or hospital first in spite of being more expensive.

The most common reasons cited for the above are reflective of inefficient care coordination and referral systems between the various levels of healthcare dealing with disability rehabilitation. Also evident is the dearth of information among general population as well primary health care providers about the various institutions providing specialized care which leads to the delay in seeking appropriate rehabilitative care. From the pattern of subsequent consultations it is clear that the parents had to go through a circuitous route to ultimately reach the specialized care centres. This delay and harassment could have been avoided if proper mechanisms were in place to channelize them earliest to the appropriate centres. Similar results were found in another study by Rosenberg *et al.* in which 25.2% of families of disabled children had to face the lack of care coordination and poor link-up between the basic care with rehabilitative care.(5)

In this study, there was a significant statistical association of the socio- economic status with the place of first consultation with an OR =0.24 (95% C.I 0.09-0.63) χ^2^ =of 10.68 and *P* value of 0.00108. A study by Yu S.M *et al*. showed parents of disabled children, with lower socio-economic status more likely to have an unmet need for treatment and care services (OR=1.88).(6) Kogan *et al.* found that even in developed countries, the low-income families with disabled children were more likely to report difficulty in obtaining specialty referrals and experienced financial problems.(7)

Although the median time lapse between onset of disease and first consultation was six hours, the median time lapse between onset of disease and commencement of the treatment or rehabilitative therapy was one month. This highlights the fact that the commencement of treatment in majority of cases is delayed inspite of timely consultation. No significant association was found between the median time lapse and the grade of disability in the present study. While in another study, the grade of severity of disability were significantly associated with lack of ancillary services, delay in therapy and inadequate communication among providers (OR = 1.14, OR = 1.72, OR = 2.45, and OR = 3.08, respectively).(8)

## Conclusions

The findings indicate that the treatment seeking behavior, particularly the first consultation of the parents of disabled children, was poor. The lower socio-economic status and place of residence were significantly associated with the treatment seeking pattern. Substantial gaps exited in the knowledge about nearby rehabilitative and counseling facilities by the parents and primary providers which lead delay in seeking appropriate and timely rehabilitative care. So there is a need for care-coordination and smooth referral link-ups among the various levels of health service providers, particularly those providing physiotherapy, fitting aids and appliances.

## Recommendations

Awareness generation and updated information dissemination at all levels should be undertaken for not only for parents but also the services providers at all levels. This can be done by conducting trainings for grass-root level workers, CMEs and inclusion of rehabilitative care in the medical curriculum. Community Based Organizations and NGOs can be linked-up with the regional level and national institutes through public-private partnership in order to strengthen the case-finding timely detection and referral systems. Schemes of ADIP (Assistance to Disabled Persons for purchase or fitting of Aids and Appliances) should be popularized and implemented through agencies like NGOs, ALIMCO (Artificial Limb Manufacturing Corporation of India), Zilla Panchayats, District Disability Rehabilitation Centre, Red Cross Society etc which will in turn improve the treatment-seeking behavior.

## References

[1] (2002). Report No. 485, Ministry of Statistics and Program Implementation NSSO National Sample Survey Organisation, 58th Round.

[2] American Academy of Pediatrics Council on Children with Disabilities (2005). ‘Care coordination in the medical home: integrating health and related systems of care for children with special health care needs’. Pediatrics.

[3] Thyen U, Sperner J Unmet (2003). Health care needs and impact on families with children with disabilities in Germany. Ambulatory Pediatric J.

[4] Agarwal R, Raina R (1991). Handbook on Disability Rehabilitation.

[5] Rosenberg D, Onufer C, Clark G, Wilkin T, Rankin K, Gupta K (2005). The need for care coordination among children with special health care needs in Illinois. Matern Child Health J.

[6] Yu SM, Nyman RM, Kogan MD, Huang ZJ, Schwalberg RH (2004). Parent's language of interview and access to care for children with special health care needs. Ambul Pediatr.

[7] Kogan MD, Newacheck PW, Honberg L, Strickland B (2005). Association between under-insurance and access to care among children with special health care needs in the United States. Pediatric.

[8] Stein RE, Silver EJ (2005). Are rates of functional limitations associated with access to care? A state-level analysis of the national survey of children with special health care needs. Matern Child Health J.

